# Identifying Women at Risk for Polycystic Ovary Syndrome Using a Mobile Health App: Virtual Tool Functionality Assessment

**DOI:** 10.2196/15094

**Published:** 2020-05-14

**Authors:** Erika Marie Rodriguez, Daniel Thomas, Anna Druet, Marija Vlajic-Wheeler, Kevin James Lane, Shruthi Mahalingaiah

**Affiliations:** 1 Department of Environmental Health Harvard TH Chan School of Public Health Boston, MA United States; 2 Department of Obstetrics and Gynecology Boston University School of Medicine Boston, MA United States; 3 Clue BioWink GmbH Berlin Germany; 4 Department of Environmental Health Boston University School of Public Health Boston, MA United States; 5 Division of Reproductive Endocrinology and Infertility Department of Obstetrics and Gynecology Massachusetts General Hospital | Harvard Medical School Boston, MA United States

**Keywords:** polycystic ovary syndrome, mobile health app, Clue, menstrual irregularities, telemedicine, mHealth, mobile phone

## Abstract

**Background:**

Polycystic ovary syndrome (PCOS) is an endocrine disrupting disorder affecting about 10% of reproductive-aged women. PCOS diagnosis may be delayed several years and may require multiple physicians, resulting in lost time for risk-reducing interventions. Menstrual tracking apps are a potential tool to alert women of their risk while also prompting evaluation from a medical professional.

**Objective:**

The primary objective of this study was to develop and pilot test the irregular cycle feature, a predictive model that generated a PCOS risk score, in the menstrual tracking app, Clue. The secondary objectives were to run the model using virtual test subjects, create a quantitative risk score, compare the feature’s risk score with that of a physician, and determine the sensitivity and specificity of the model before empirical testing on human subjects.

**Methods:**

A literature review was conducted to generate a list of signs and symptoms of PCOS, termed variables. Variables were then assigned a probability and built into a Bayesian network. Questions were created based on these variables. A total of 9 virtual test subjects were identified using self-reported menstrual cycles and answers to the feature’s questions. Upon completion of the questionnaire, a Result Screen and Doctor’s Report summarizing the probability of having PCOS was displayed. This provided information about PCOS and data to facilitate diagnosis by a medical professional. To assess the accuracy of the feature, the same set of 9 virtual test subjects was assigned probabilities by the feature and the physician, who served as the gold standard. The feature recommended individuals with a score greater than or equal to 25% to follow-up with a physician. Differences between the feature and physician scores were evaluated using a t test and a Pearson correlation coefficient in 8 of the 9 virtual test subjects. A second iteration was conducted to assess the feature’s probability capabilities.

**Results:**

The irregular cycle feature’s first iteration produced 1 false-positive compared with the physician score and had an absolute mean difference of 15.5% (SD 15.1%) among the virtual test subjects. The second iteration had 2 false positives compared with the physician score and had an absolute mean difference of 18.8% (SD 13.6%). The feature overpredicted the virtual test subjects’ risk of PCOS compared with the physician. However, a significant positive correlation existed between the feature and physician score (Pearson correlation coefficient=0.82; *P*=.01). The second iteration performed worse, with a Pearson correlation coefficient of 0.73 (*P*=.03).

**Conclusions:**

The first iteration of the feature outperformed the second and better predicted the probability of PCOS. Although further research is needed with a more robust sample size, this pilot study indicates the potential value for developing a screening tool to prompt high-risk subjects to seek evaluation by a medical professional.

## Introduction

### Background

According to the Rotterdam criteria, polycystic ovary syndrome (PCOS) is clinically diagnosed by the presence of at least two of the following: androgen excess, menstrual irregularity, or presence of polycystic ovary morphology on ultrasound examination [1]. The Androgen Excess and PCOS Society has also proposed guidelines for diagnosis, which include hyperandrogenism, ovarian dysfunction, and exclusion of other androgen excess or related disorders [2]. Women with menstrual irregularities, particularly those with PCOS, have an increased risk of developing comorbidities, such as metabolic syndrome, heart disease, or diabetes [3]. In 2005, the economic burden of evaluating and providing care to women of reproductive age with PCOS was US $4.36 billion, which was equivalent to US $5.65 billion in 2018 [4]. This assessment included the costs of infertility treatments, living and treating metabolic disorders, and addressing hirsutism. The calculated expenses of PCOS are likely an underestimate of the actual cost of providing care because many women will live beyond reproductive age with expensive metabolic disorders such as metabolic syndrome, which adds at least US $2000 more of health care expenses annually [4,5].

In the current era of ubiquitous smartphones, individuals are turning to mobile phone apps for immediate health tracking and care [6,7]. Women, in particular, have reported higher rates of downloading health apps [8]. By having menstrual cycle data collected in real time, women are able to share accurate details with their medical providers [9]. Thus, these apps can have significant implications for women with menstrual irregularities.

For women at high risk of developing PCOS, having a mobile health app that identifies and tracks menstrual cycles may help facilitate symptom tracking and prevent inaccurate reporting of signs and symptoms [10]. However, current menstrual tracking apps are not well equipped to deal with irregular menstrual cycles [11].

### Objectives

The primary objective was to develop and pilot test the irregular cycle feature. The irregular cycle feature is a predictive model that generates a PCOS risk score for a user based on responses to an adaptive questionnaire. The secondary objectives were to run the model using 9 virtual subjects, create a quantitative risk score, compare the model’s risk score with that of a board-certified reproductive endocrinology and infertility physician-scientist (physician), and determine the sensitivity and specificity of the model before empirical testing on human subjects.

## Methods

### Creating the Framework

Clue is an app created by BioWink GmbH, which allows users to track menstrual cycles and related health information [12]. Data collected include, but are not limited to, menstrual cycle length, duration of flow, menstruation-related pain symptoms, and method of birth control. To create a user profile, the app also prompts individuals to input age, height, and weight. Personally identifiable information is collected by Clue and stored in a secure backend system where it is encrypted [13].

**Figure 1:**
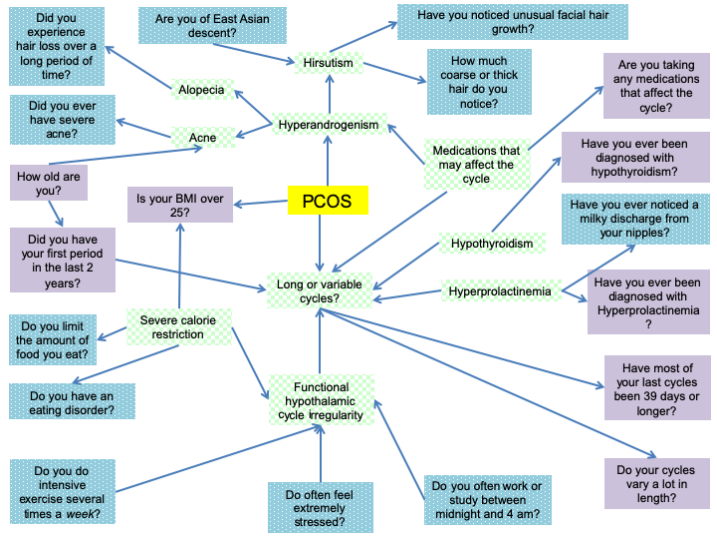
Green, checkered boxes indicate major diagnostic concerns for polycystic ovary syndrome. Purple, solid boxes are representative of the questions that are on the screen. The blue, dotted boxes are additional questions asked by the irregular cycle feature. PCOS: polycystic ovary syndrome.

An adaptive questionnaire, or a question set modified based on answers to previous questions, was developed. To create the framework for the adaptive questionnaire, the physician from Boston University and researchers at Clue compiled signs and symptoms (variables) associated with PCOS based on the Rotterdam criteria and common diseases that display similar variables based on the physician’s clinical experience. For the purpose of developing this tool, other common diseases accounted for in the Bayesian network created with overlapping signs and symptoms included Cushing syndrome, hypothyroidism, hyperprolactinemia, and functional amenorrhea. The physician developed a list of common variables for these disorders based on clinical experience and the Rotterdam diagnostic criteria [1]. These variables included irregular menstrual cycles, hirsutism, alopecia, and acne. A literature review was then conducted to determine the prevalence of these variables specifically in women with PCOS. The literature review consisted of searches in UpToDate (Wolters Kluwer Health’s) for the prevalence of each included variable. When a search yielded no prevalence, an additional search was conducted on PubMed to determine the appropriate prevalence. For example, the team’s PubMed search using the MeSH term *hyperandrogenism* for clinical hyperandrogenism yielded a 10% prevalence in women of reproductive age [14]. Each variable and associated prevalence were then incorporated into a Bayesian network to generate joint probabilities (Figure 1). A total for 40 unique articles were used to determine the prevalence for the 4 variables

A Bayesian network is a modeling tool that integrates independent and dependent probabilities to calculate an overall probability based on each variable [15]. The Bayesian network was created manually using Netica. For each variable, the prevalence of symptoms, given the presence or absence of a disease, was manually encoded in the Bayesian network. The prevalence of each symptom determined in the literature review was then inserted as the probability for each variable. The physician was consulted when published values were widely variable. For nodes in the network with many parents, such as *long or variable cycles*, there were many possible combinations of dependencies. For simplicity, they were assumed to be uncorrelated. The software tool then constructed the Bayesian network. Figure 1 is reflective of the Bayesian network that was generated using the Netica software with probabilities omitted. Specific parameters for each variable are proprietary to Clue.

### Phase 1: Development of Question Sets

#### Virtual Test Subjects

A virtual test subject is an algorithmic model representative of how an individual or a group of individuals may appear or behave in the real world [16]. These computer models have proven useful in creating predictive models for epidemics, such as the spread of influenza or serving as educational materials for physicians [17,18]. Virtual test subjects have also previously been used to train physicians on the administration of glucose clamp procedures [16]. To conduct the pilot testing of this feature, a set of virtual test subjects was created. Each virtual test subject was generated by a designated team member and had a unique set of answers to preformulated questions that were built into the irregular cycle feature. For example, 1 virtual test subject would be asked whether or not they experienced excess hair growth and would respond yes, whereas a different virtual test subject would respond no. These slight changes in responses allowed the team to run an analysis to understand the validity of the network. The scores of the irregular cycle feature generated for each of these virtual subjects were then compared with the score provided by a physician. The answers to each question for all virtual test subjects remained consistent between the irregular cycle feature and physician scoring. Only 14 virtual test subjects were created for ease of testing the feature.

#### Eligibility Requirements

The inclusion criteria, which were based on the definitions of abnormal uterine bleeding from the International Federation of Gynecology and Obstetrics, for prompting with the irregular cycle feature included logging irregular cycles for at least 6 months or have not logged a period in 90 days, cycles longer than 38 days, or a cycle length variation that is higher than average for their age group. For individuals aged between 16 and 26 years, the cycle length variation threshold is >9 days cycle to cycle. For users aged >27 years, the cycle length variation should be >7 days [19]. A virtual test subject would be excluded from the study if these criteria were not met.

#### Screener Questions

Once the network was established, questions were written to assess signs and symptoms deemed relevant by the literature review. Questions were separated into a screener and the adaptive questionnaire, within the irregular cycle feature. The screener consisted of 7 questions used to collect information regarding height, weight, age, birth control use, medical conditions, life stage, and age at menarche. The specific questions and abbreviated answers are shown in Multimedia Appendix 1.

#### Adaptive Questionnaire

After the screener, the subject was presented with the adaptive questionnaire, which features questions aimed at completing the picture of risk factors for PCOS. These questions were based on the Ovulation and Menstruation Health Study conducted at Boston University [20], which measures androgen excess, levels of stress, and eating patterns. Questions in the adaptive survey portion of the irregular cycle feature included measures of body hair, medications that can affect the menstrual cycle, hair loss, acne, irregular sleep, stress, strenuous physical work, and eating habits. Although this was not an exhaustive medical history, these variables were deemed useful for preliminary identification of PCOS based on the current literature. A complete list of additional questions assessed by the feature is shown in Multimedia Appendix 2. As some symptoms vary by racial and ethnic groups, virtual test subjects were also able to report their background. In total, 14 virtual test subjects were created to assess the functionality of the Bayesian network. Each of the virtual test subjects had unique answers to the irregular cycle feature screener and the adaptive questionnaire to test the ability of the network to produce an accurate PCOS probability. Each virtual test subject had a different combination of questions left blank. Leaving questions blank allowed the team to determine how accurate the irregular cycle feature could score virtual test subjects compared with the physician in the setting of missing data. The questions left blank were selected based on the relative missing data that currently exist in the user-entered data in the Clue app. The 14 virtual test subjects created were generated to be comparable with aggregated, deidentified Clue user data. Nine virtual test subjects were considered representative of cycles, birth control usage reporting, and symptom tracking available from the Clue user base and were included in the analysis.

### Phase 2: Question Flow

**Figure 2:**
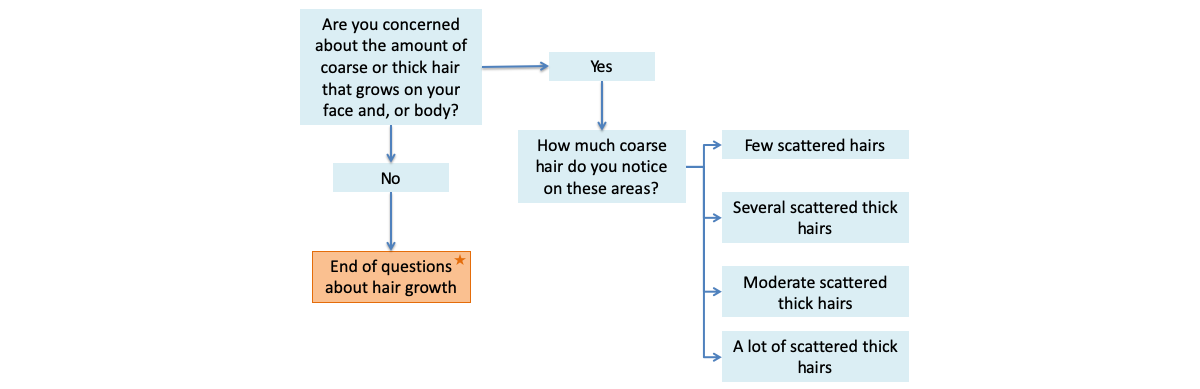
This figure highlights the adaptive nature of irregular cycle feature by modeling with questions assessing hair growth. The orange, starred box indicates the end of the question set.

As the irregular cycle feature is adaptive, not every question is asked to every subject. For instance, Figure 2 shows that if a virtual test subject reported that they were not concerned about excess hair growth, the tool would not ask about how much hair was present on their body. In this manner, the model streamlined the data collection and reduced the time it took a subject to complete the module. Compiling this information, the network then calculated whether menstrual cycle irregularities were an indicator of PCOS or if the menstrual irregularities were possibly because of a different condition.

### Phase 3: Assignment of Thresholds for Risk

The physician on the team assigned 4 categories of risk: low, indeterminate, moderate, or high to each virtual test subject based on her assessment of the virtual test subject’s symptoms compared with the Rotterdam diagnostic criteria for PCOS. According to the Rotterdam criteria, PCOS is clinically diagnosed by the presence of at least two of the following: androgen excess, menstrual irregularity, or presence of polycystic ovary morphology on ultrasound examination [1]. TThe physician was shown the responses from each of the virtual test subjects that were run through the irregular cycle feature. She then determined a category of risk based on the Rotterdam diagnostic criteria excluding hypothalamic and thyroid causes based on responses to questions. For example, if a virtual test subject presented with increased body hair but also reported a history of disordered eating, the body hair was more likely to be associated with anorexia than with PCOS. The physician would then assign a low risk category. To compare this with the quantitative values generated by the irregular cycle feature, the team determined a set of numerical ranges for the categories based on proposed definitions for converting qualitative and quantitative classifications by Hillson [21].

**Figure 3:**
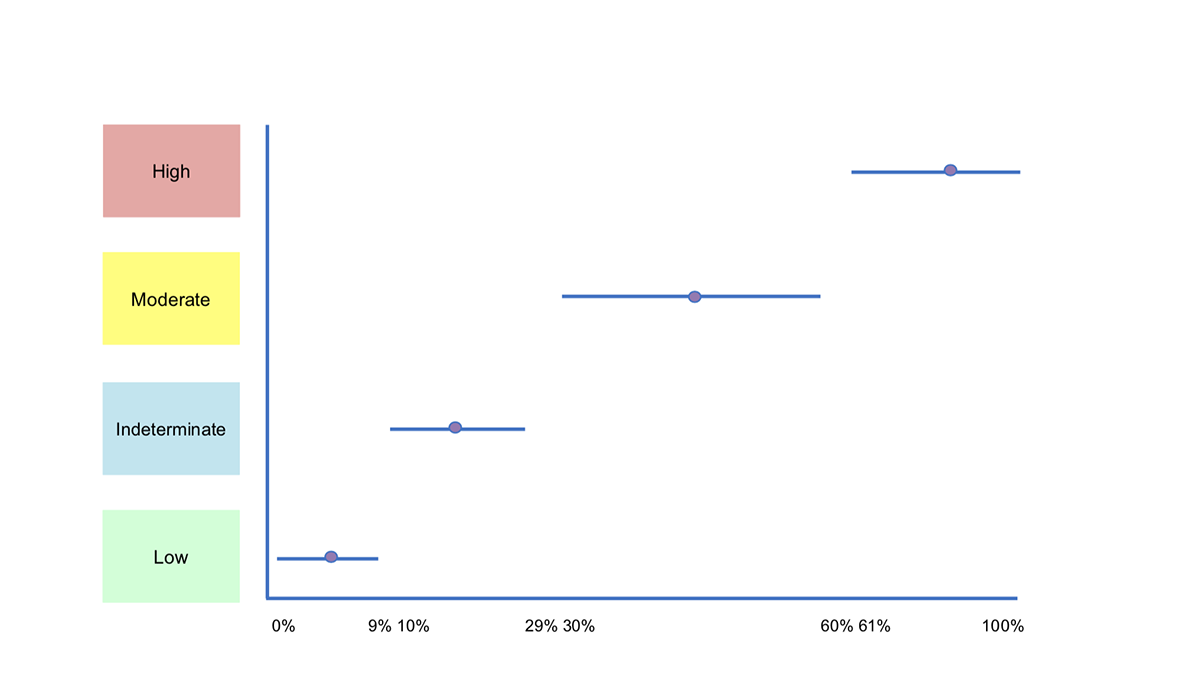
Sliding scale percent probability ranges. The blue bar indicates the range of percentages that fall into the high, moderate, indeterminate, and low categories. The purple circle illustrates the percentage typically used in the assignment of percent probability.

Virtual test subjects that were determined to have a low risk of PCOS were assigned a value within the range of 0% to 9%. The numerical range for indeterminate risk was assigned as 10% to 29%. These values were selected based on the *unlikely* and *possible* qualitative terms in the proposal by Hillson [21]. Virtual test subjects with moderate individual risk were assigned percentages within the range of 30% to 59%. These values encompassed the categories of *probable* and *good chance* as defined by Hillson [21]. Virtual test subjects with high individual risk were assigned a value within the range of 60% to 100%. These values were selected based on the Hillson’s interpretation of *highly probable* to *definite* terms [21]. These probability scales are summarized in Figure 3.

During the evaluation of the virtual test subjects, the physician determined that 2 virtual test subjects were confounded by additional variables and could not be accurately assessed because of lack of data. These 2 cases were assigned a probability of 10%, as they fell into the indeterminate category. A total of 10% was arbitrarily chosen for this study because it is reflective of a low but possible risk of developing PCOS.

Results Screens were created to provide feedback at the end of the assessment based on each of these thresholds. Distinct and direct recommendations were given based on the calculated probability of PCOS yielded by the irregular cycle feature. There were 3 possible screens, including a Positive Result Screen, a Neutral Result Screen, and an Inconclusive Result Screen. All screens include a brief description of how the result was reached and a disclaimer that the result was not a diagnosis.

Users calculated as having a probability of PCOS that was greater than or equal to 25% by the irregular cycle feature were prompted with a Positive Result Screen (Multimedia Appendix 3). This screen displays a description of PCOS, related health risks, and a call to action encouraging the user to seek medical attention. In addition, it described the steps a physician may take to diagnose the individual with PCOS or another related disorder affecting menstrual cycle regularity.

The Neutral Result Screen (Multimedia Appendix 4) was presented to users with an irregular cycle feature probability of less than 25%. It stated that a prediction could not be made regarding what was causing the irregularities. It included other potential causes such as lifestyle factors, hypothyroidism, and Cushing syndrome. The user was also prompted to seek advice from a medical professional. The text described that the physician would likely perform a detailed history regarding symptoms, a simple physical examination, and blood tests if necessary.

The Inconclusive Screen (Multimedia Appendix 5) was presented to users who have reported confounding variables or too much missing data. These variables included, but were not limited to, the use or recent discontinuation of hormonal birth control, age outside applicable range, recent pregnancy, and breastfeeding. The potential use of medications, particularly those with hormones, in individuals creates too many confounding variables and is beyond the capabilities of the network’s calculations. Thus, when an individual reported a confounding variable that also causes hormonal dysregulation, the screen prompted them with a suggestion to visit a medical professional who can perform additional testing to determine the reason behind their menstrual irregularity. Of the 14 virtual test subjects, 6 were ultimately prompted with an Inconclusive Screen because of their answers.

The Doctor’s Report was a shareable document that was generated at the end of each assessment for presentation to a medical professional (Multimedia Appendices 6 and 7). It included details regarding the virtual test subject’s menstrual cycle characteristics and history as well as the signs and symptoms reported via the irregular cycle feature questionnaire. The medical board at Clue and the physician from Boston University were consulted in its design to ensure that it appropriately highlighted a user’s health data. The Doctor’s Report was generated to determine whether the individual was expected to have PCOS or not. This ensured that the individual was able to provide important medical information to their provider regardless of the output so long as their cycles were irregular.

### Phase 4: Validation of the Tool

To validate the usability and accuracy of the network, the physician-generated probabilities from phase 3 were compared with those predicted by the irregular cycle feature. This helped determine whether the feature could make assessments similar to those performed in a clinical setting. A summary of the probability assignments by both the irregular cycle feature and the physician is shown in Table 1.

**Table 1:** Probability assignments^a^

Virtual test subject #	Physician’s assessment	Physician’s assigned probability, %	Irregular cycle feature’s assigned probability, %
Subject 1	High	80	91
Subject 2	High	80	93
Subject 8	PCOS^b^: 30%; hypothalamic pituitary: 70%	30	66
Subject 12	Currently taking hormones: this is a special case—indication of irregular cycles means the feature should direct them to talk to their physician	10	10
Subject 14	Low PCOS	5	34
Subject 17	Moderate to high PCOS	70	37
Subject 5	Indeterminable	10	9
Subject 7	High	80	14
Subject 15	None	0	1

^a^Summary of the physician’s assessments (categorical probabilities), physician’s assigned probabilities, and the irregular cycle feature calculated probability for all test subjects that went through the irregular cycle feature questionnaire during the first iteration.

^b^PCOS: polycystic ovary syndrome.

### Statistical Analysis

Microsoft Excel version 16.16 was used to conduct all statistical analyses. The utility of the irregular cycle feature was assessed by (1) comparing the sensitivity (false-negatives) and specificity (false-positives) with the assessment made by the physician, (2) mean difference among virtual test subject scores, and (3) the Pearson correlation coefficient. Normality was assessed by plotting the data points. The correlation between the irregular cycle feature and physician, a *P* value from the correlation, and a Pearson correlation coefficient were calculated using the 9 eligible virtual test subjects. Multimedia Appendix 8 illustrates the sensitivity, specificity, and mean difference for the 9 virtual test subjects. A second analysis was then conducted, excluding 1 virtual test subject. When the outlier data point was removed, there was a normal distribution in the scores. As this data point significantly skewed the values, the data resulted in a final set of 8 virtual test subjects that were used to assess the sensitivity and specificity, mean difference, and Pearson correlation coefficient.

An additional iteration of the irregular cycle feature’s Bayesian network was also completed to determine the effect of hirsutism on the accuracy of scoring for PCOS in the virtual test subjects. To assess whether the prevalence previously selected for hirsutism was reflective of current literature, a second literature review was conducted. This review yielded a slightly lower prevalence of hirsutism in PCOS. As such, this new prevalence was integrated into the irregular cycle feature’s Bayesian network. The virtual test subjects were run through the feature again. A summary of the statistical tests and outcomes for the old and new prevalence can be seen in Tables 2-5.

**Table 2:** Summary of statistical calculations for 9 virtual test subjects.^a^

Statistical test	Value
Pearson correlation coefficient	0.62
*P* value	.08

^a^Statistics for all virtual test subjects that were created by the team, including subject 7, who was ultimately excluded because it was a statistical outlier.

**Table 3:** Summary of statistical calculations for 8 virtual test subjects.^a^

Statistical test	Value
Pearson correlation coefficient	0.82
*P* value	.01

^a^Statistics for 8 test subjects created by the team. It excludes subject 7, who was suspected to be an outlier based on the mean difference.

**Table 4:** Summary of statistical calculations for 8 virtual test subjects on the second iteration of the irregular cycle feature.^a^

Statistical test	Value
Pearson correlation coefficient	0.73
*P* value	.03

^a^Statistics calculated with lowered probabilities of hirsutism in polycystic ovary syndrome for all test cases excluding subject 7 (the suspected outlier).

**Table 5:** Sensitivity, specificity, and mean difference of irregular cycle feature and physician scores by iteration.

Virtual test subject		Feature (%)	Physician (%)	Specificity	Sensitivity	Difference (%)
**Iteration number 1^a^**						
	1	91	80	0	0	11
	2	93	80	0	0	13
	8	66	30	0	0	36
	12	10	10	0	0	0
	14	34	5	1	0	29
	17	37	70	0	0	−33
	5	9	10	0	0	−1
	15	1	0	0	0	1
**Iteration number 2^b^**						
	1	88	80	0	0	8
	2	92	80	0	0	12
	8	56	30	0	0	26
	12	10	10	0	0	0
	14	26	5	1	0	19
	17	29	70	0	0	−31
	5	22	10	0	0	12
	15	42	0	1	0	42

**^a^**Absolute mean difference for iteration 1 was 15.5% (SD 15.1%); mean difference 7%.

**^b^**Absolute mean difference for iteration 1 was 18.8% (SD 13.6%); mean difference: 11%.

## Results

**Figure 4:**
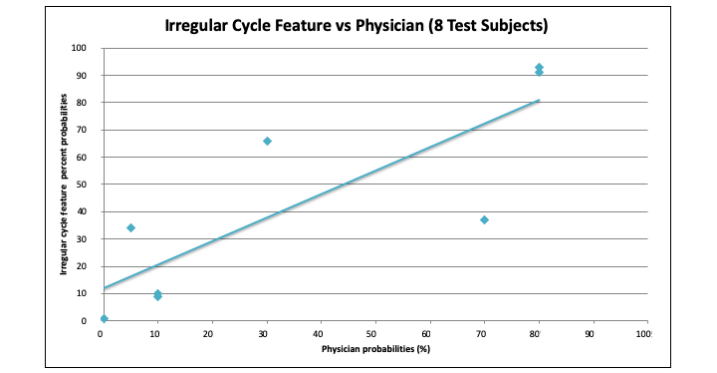
This graph demonstrates the linear regression for 8 test cases, excluding subject 7 (the suspected outlier).

The majority of virtual test subjects had an irregular cycle feature score that overpredicted PCOS when compared with the physician screening score (Table 5). The first iteration of the irregular cycle feature produced only 1 false-positive compared with the physician screening score and had an absolute mean difference of 15.5% (SD 15.1%) among virtual test subjects. The second iteration of the irregular cycle feature had 2 false positives compared with the physician screening score and had an absolute mean difference of 18.8% (SD 13.6%). The first iteration irregular cycle feature score overpredicted the probability of PCOS compared with the physician with a mean absolute difference of 7. The correlation value was calculated to be 0.82 for the 8 virtual test subjects. The same 8 virtual test subjects were also used to generate a linear regression (Figure 4). The *P* value was then determined to be .01 (Table 3). As the *P* value was less than .05, the team determined that the predictive capabilities of the irregular cycle feature were statistically significantly different from the assessments made by the physician, although the risk difference was not considered to be clinically significant. A few sample virtual test subjects are shown in Table 6 to demonstrate the questions, answers, and predictions generated by the irregular cycle feature.

The results generated by the irregular cycle feature compared with those assigned by the physician are shown in Table 1. For virtual test subject 1, the physician assigned an 80% probability, compared with the 91% probability calculated by the feature. In both cases, the virtual test subject would be prompted with a Positive Result Screen and the Doctor’s Report stating PCOS as a possible cause for irregular menstrual cycles.

In virtual test subject 8, the physician assigned a 30% probability of PCOS and further suggested a hypothalamic or pituitary cause of the menstrual irregularities. The irregular cycle feature, which predicted a 66% probability, would prompt the individual to seek advice from a medical professional via the Positive Result Screen and also suggest PCOS as a possible cause for signs and symptoms reported.

For virtual test subject 12, the physician assigned a 10% probability of PCOS. The irregular cycle feature also suggested a 10% probability of PCOS. The physician determined that the hormones taken by the individual indicated a special case, and that recommendations could not be made based on the answers provided. The feature’s Inconclusive Screen was presented to this virtual test subject.

For virtual test subject 15, both the physician and the irregular cycle feature predicted a 0% to 1% chance of PCOS, and the subject received the Neutral Result Screen and Doctor’s Report stating PCOS as an unlikely cause of menstrual irregularities.

**Table 6:** Sample questions, answers, and probabilities using irregular cycle feature.^a^

Question	Subject 1	Subject 8	Subject 12	Subject 15
Concerned about hair growth?	Yes	Yes	Yes	No
How much thick hair?	Lots of Hair	Several	Several	—^b^
Majority of cycles ≥38 days?^c^	Yes	Yes	Yes	No
Taking meds that could affect the cycle?	No	No	Yes	—
BMI >25 kg/m^2^^c^	No	No	No	—
Cycle variation out of range?^c^	Yes	—	—	No
Eating disorder?^c^	No	Yes	—	—
Hair loss?	No	No	—	—
Acne?	No	No	—	—
Menarche during last 2 years?^c^	No	—	—	—
Age range?^c^	—	19-40	—	—
Of East Asian heritage?^c^	—	—	—	—
Diagnosed with hypothyroidism?^c^	—	—	—	—
Irregular sleep?	—	—	—	—
Stress?	—	—	—	—
Strenuous physical work?	—	—	—	—
Limiting food?	—	—	—	—
Diagnosed with hyperprolactinemia?	—	—	—	—
Polycystic ovary syndrome probability^d^	91%	66%	10%	1%

^a^This table displays the virtual test subject answers to the irregular cycle feature questionnaire

^b^Data not included in a virtual test subject’s answer (ie, sections left blank)

^c^Screener questions.

^d^Polycystic ovary syndrome percent probability generated by the irregular cycle feature

## Discussion

### Principal Findings

To the best of our knowledge, this is the first app developed by an interdisciplinary team to calculate the probability that an individual may have a risk of PCOS. As seen in virtual test subject 1, the similarly high probabilities assigned by the physician and irregular cycle feature demonstrate that in textbook cases of PCOS, the feature accurately prompts individuals to seek out a medical professional. This will be important for the identification of several risk factors for PCOS. Virtual test subject 8 highlights the overpredictive nature of the irregular cycle feature. Although the irregular cycle feature probability is slightly higher than the physician’s prediction, the tool still proves useful, as it advises an individual to seek a health care provider for further testing. In addition, the lack of data input for virtual test subject 8, specifically for menstrual variation and confounding diagnoses, can be improved once data are collected from actual Clue users to make more accurate predictions. Virtual test subject 12, who used hormonal birth control, demonstrates how the feature calculates a probability for individuals inputting the minimum amount of information and confounders while also indicating menstrual irregularities. The irregular cycle feature would prompt similar individuals to seek a medical professional for any issues regarding their menstrual irregularities. Virtual test subject 15 did not report menstrual irregularities nor did they indicate areas of concern, such as those associated with hyperandrogenism: acne, alopecia, or hirsutism. This illustrates that the model can accurately eliminate individuals who are unlikely to have a disorder.

### Strengths and Limitations

The team was composed of data and health scientists, software engineers, and a medical expert. Together we constructed a mobile health tool to facilitate identification of possible indicators of PCOS in an app-using population. Furthermore, the irregular cycle feature allows users to self-report menstrual information to facilitate discussion with their physicians. Since a summary generated based on the answers to the interactive survey is specific to each individual, more control is in the hands of the user. In addition, by providing the user with an outline of what the next steps are in terms of testing and visiting a medical professional’s office, on rollout to Clue users, this novel tool could shorten the time for a PCOS diagnosis.

The limitations of this study include (1) the use of only a small number of virtual test subjects, (2) the lack of a clinical validation, and (3) the limited applicability of the feature to other disorders that alter menstrual cycle regularity. The small number of virtual test subjects eligible for the irregular cycle feature assessment allowed for modeling how predictive the tool could be with a significant number of missing data points. In a setting where all data points are captured, the feature may have better predictability. Furthermore, because of the nature of this pilot project, the tool has not yet been clinically validated in a human population, and thus, the probability generated by the tool is not a diagnosis. At present, the model cannot make predictions for individuals who report the use of hormone-based medications or facilitate risk prediction for other syndromes that lead to menstrual irregularity, such as Cushing syndrome or hypothyroidism. Further limitations of this study include assigning a single quantitative value to the physician’s qualitative assignment of risk (low, indeterminate, medium, and high). Since there is currently no way to assign a range of probabilities to the physician’s qualitative score, the team was limited to assigning a single, estimated probability value. Finally, the irregular cycle feature is currently modeled to have a high sensitivity and low specificity, erring on the side of sending more individuals to the physician’s office as an attempt to capture the majority of individuals at risk for PCOS. It remains to be seen whether the predictive model will be adaptable to actual human users.

Under appropriate institutional review board approval, a future validation study of the irregular cycle feature will be necessary to assess the utility of the tool between physicians by following up with users at variable time intervals to determine if a medical professional diagnosed PCOS or another condition. Future iterations of this study will use real Clue users to assess the validity of the irregular cycle feature. In addition, other sources of data will be considered to train the network to establish improved conditional dependencies and make additional adjustments to the Bayesian network.

### Conclusions

Despite the limitations of the study, the irregular cycle feature is an example of how mobile technology may help users manage their own health and promote subject self-advocacy. By providing more information about PCOS and giving users feedback based on information they have collected, the research team predicts that this feature will provide important information to those at high risk of the disease and potentially shorten the time for diagnosis [22,23]. The creation of the irregular cycle feature may reduce the time to PCOS diagnosis and facilitate conversations between users and physicians through the Results Screens and Doctor’s Report.

## Appendix

Multimedia Appendix 1 Illustrates the questions in the irregular cycle feature screener and the associated abbreviated answer choices.

Multimedia Appendix 2 Categories to assess menstrual irregularities. Three different categories for assessing risk of PCOS were established based on the Ovulation and Menstruation Health Study. Each of the questions in the ICF questionnaire (excluding the Screener) is included in the table.

Multimedia Appendix 3 Supplemental Figure 1. The Positive Result Screen that would appear for users with a risk score of 25% or higher in the Clue app.

Multimedia Appendix 4 Supplemental Figure 2. The Neutral Result Screen that would appear for users with a risk score of 25% or lower in the Clue app.

Multimedia Appendix 5 Supplemental Figure 3. The Inconclusive Result Screen that would appear for users with confounding variables or too much missing data in the Clue app.

Multimedia Appendix 6 Supplemental Figure 4. The Doctor's Report that would appear for users who had a Positive Result Screen generated in the Clue app.

Multimedia Appendix 7 Supplemental Figure 5. The Doctor's Report that would appear for users who had a Neutral or Inconclusive Result Screen generated in the Clue app.

Multimedia Appendix 8 Sensitivity, specificity, and mean difference of irregular cycle feature and physician scores for the 9 virtual test subjects.

## Abbreviations

PCOS: polycystic ovary syndrome
