# Supplementary figures and images for: Identifying Women at Risk for Polycystic Ovary Syndrome Using a Mobile Health App: Virtual Tool Functionality Assessment
Source: JMIR Form Res. 2020 May 14;4(5):e15094. doi: 10.2196/15094 (PMC7256750; doi:10.2196/15094)

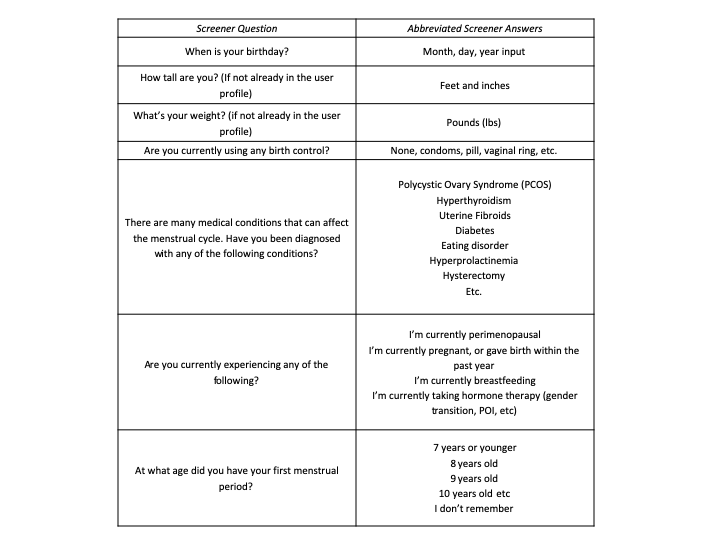

Supplement: Multimedia Appendix 1 [file formative_v4i5e15094_app1.png]

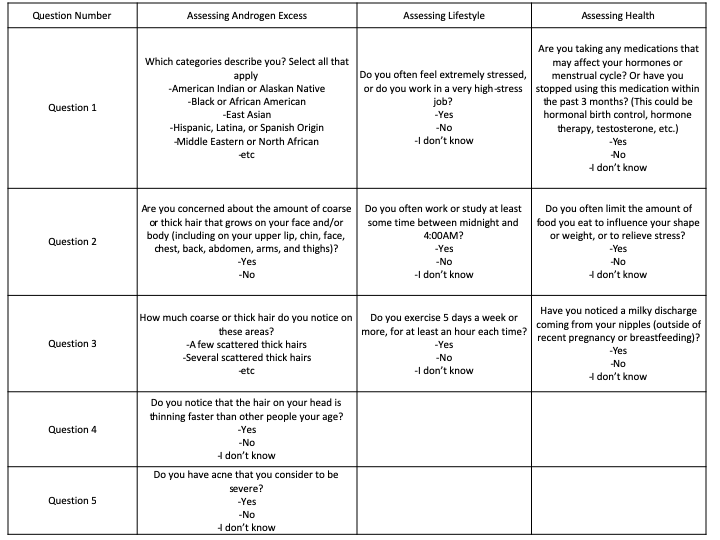

Supplement: Multimedia Appendix 2 [file formative_v4i5e15094_app2.png]

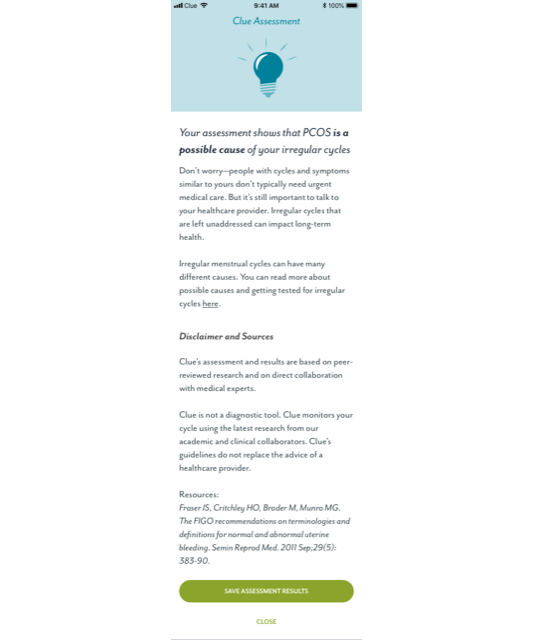

Supplement: Multimedia Appendix 3 [file formative_v4i5e15094_app3.png]

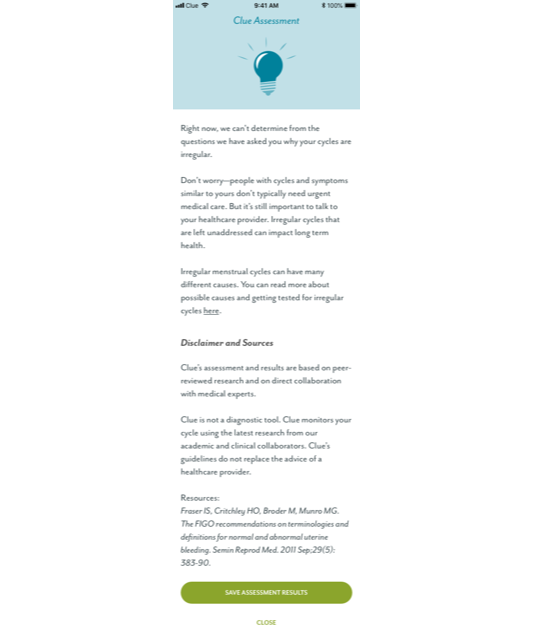

Supplement: Multimedia Appendix 4 [file formative_v4i5e15094_app4.png]

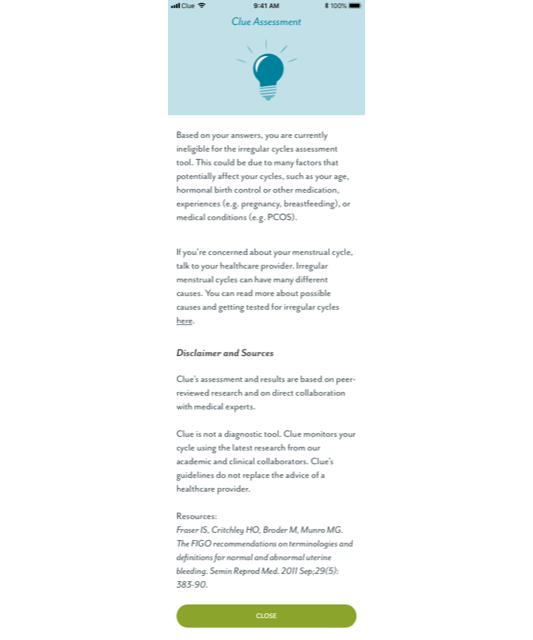

Supplement: Multimedia Appendix 5 [file formative_v4i5e15094_app5.png]

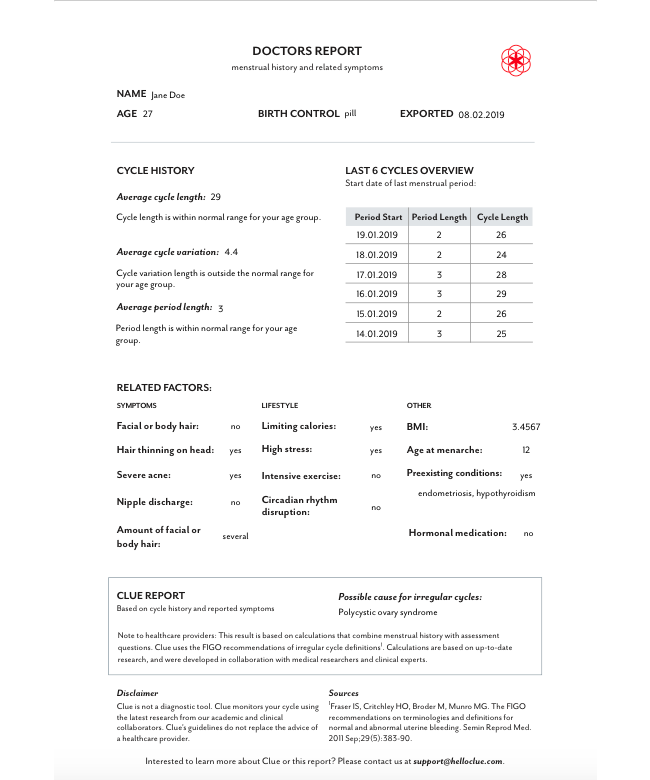

Supplement: Multimedia Appendix 6 [file formative_v4i5e15094_app6.png]

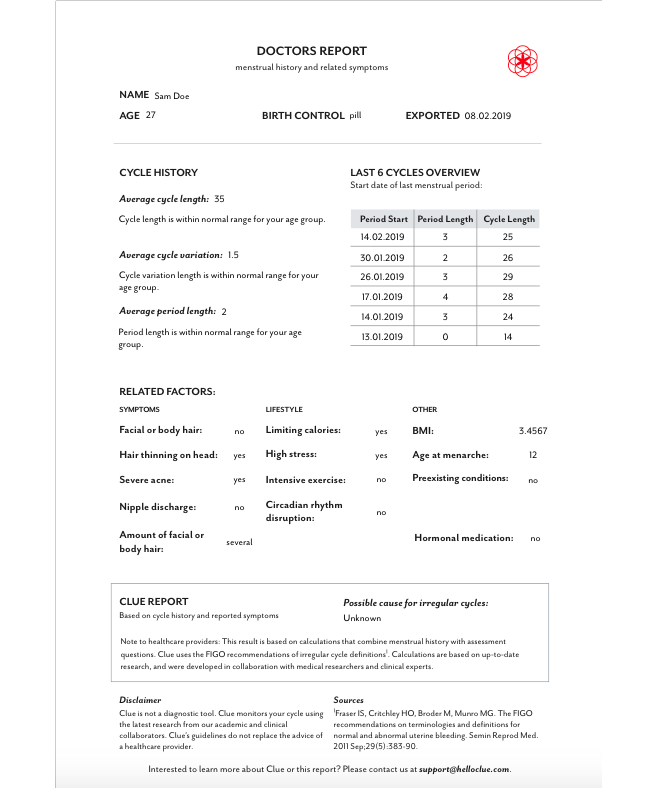

Supplement: Multimedia Appendix 7 [file formative_v4i5e15094_app7.png]

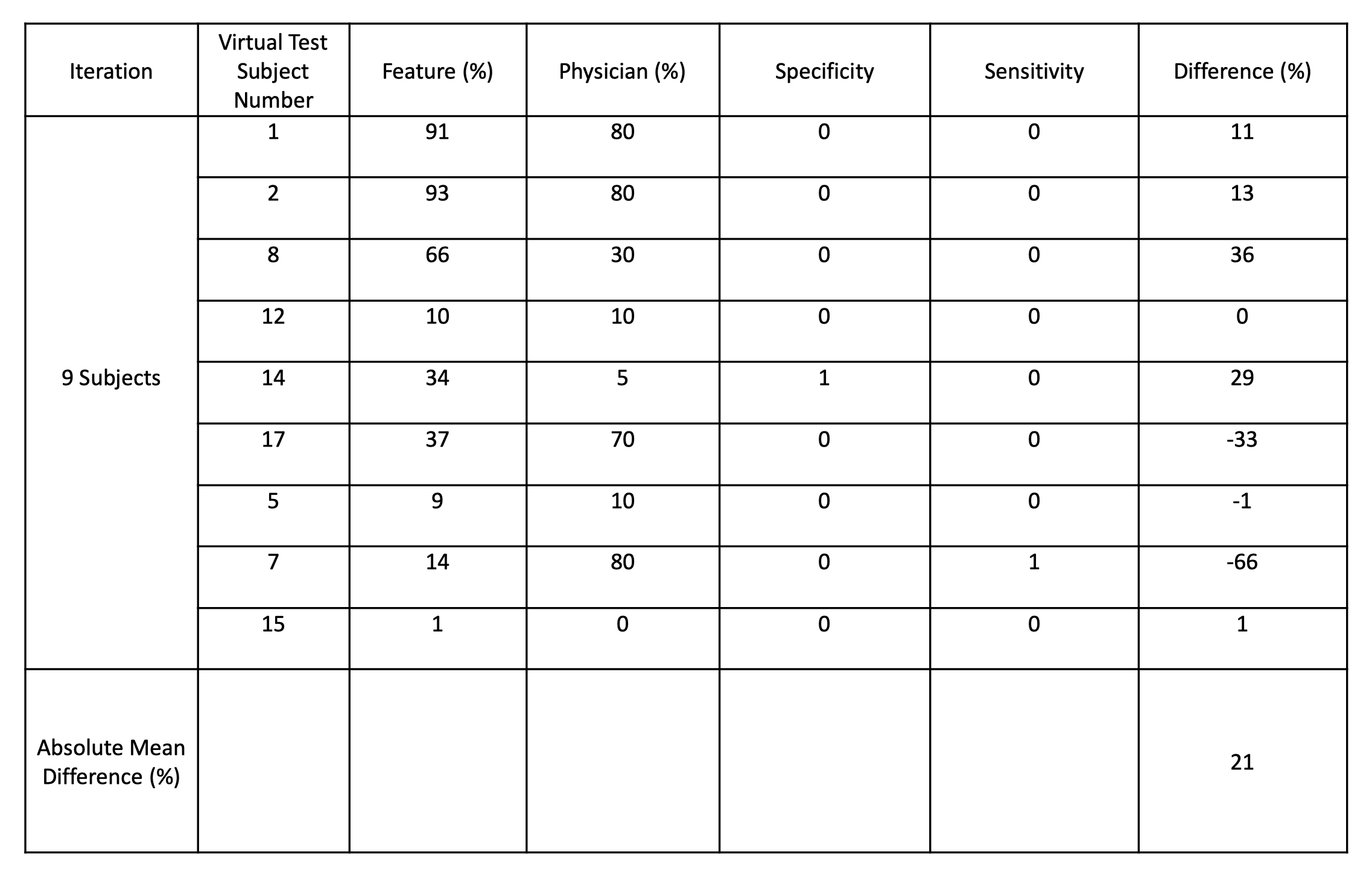

Supplement: Multimedia Appendix 8 [file formative_v4i5e15094_app8.png]
